# Protein Abundance of Clinically Relevant Drug Transporters in The Human Kidneys

**DOI:** 10.3390/ijms20215303

**Published:** 2019-10-24

**Authors:** Stefan Oswald, Janett Müller, Ute Neugebauer, Rita Schröter, Edwin Herrmann, Hermann Pavenstädt, Giuliano Ciarimboli

**Affiliations:** 1 Department of Clinical Pharmacology, University Medicine of Greifswald, 17475 Greifswald, Germany; nettie.m@web.de; 2 Medicine Clinic D, Experimental Nephrology, University Hospital of Münster, 48149 Münster, Germany; Ute.Neugebauer@uni-muenster.de (U.N.); ritas@uni-muenster.de (R.S.); Hermann.Pavenstaedt@ukmuenster.de (H.P.); 3 Urology Clinic, University Hospital of Münster, 48149 Münster, Germany; edwin.herrmann@prosper-hospital.de

**Keywords:** transporters, human kidneys, protein expression, age, gender

## Abstract

Renal drug transporters such as the organic cation transporters (OCTs), organic anion transporters (OATs) and multidrug resistance proteins (MRPs) play an important role in the tubular secretion of many drugs influencing their efficacy and safety. However, only little is known about the distinct protein abundance of these transporters in human kidneys, and about the impact of age and gender as potential factors of inter-subject variability in their expression and function. The aim of this study was to determine the protein abundance of MDR1, MRP1-4, BCRP, OAT1-3, OCT2-3, MATE1, PEPT1/2, and ORCTL2 by liquid chromatography-tandem mass spectrometry-based targeted proteomics in a set of 36 human cortex kidney samples (20 males, 16 females; median age 53 and 55 years, respectively). OAT1 and 3, OCT2 and ORCTL2 were found to be most abundant renal SLC transporters while MDR1, MRP1 and MRP4 were the dominating ABC transporters. Only the expression levels of MDR1 and ORCTL2 were significantly higher abundant in older donors. Moreover, we found several significant correlations between different transporters, which may indicate their functional interplay in renal vectorial transport processes. Our data may contribute to a better understanding of the molecular processes determining renal excretion of drugs.

## 1. Introduction

One of the most important physiological roles of the kidneys is to contribute to the maintenance of body homeostasis, by regulating salt and water excretion and eliminating metabolism waste products and xenobiotics. To achieve these aims, the nephron, that is the functional unit of the kidneys, filters the blood in a structure called glomerulus. The composition of the filtrate is then strongly modified during its passage through the tubules system, before being excreted as urine. The cells of the renal tubules are equipped with a huge number of transport proteins, which are specifically expressed in distinct domains of the cellular plasma membrane (basolateral and apical plasma membrane) and in distinct portions of the nephron [1]. Quantitatively, most parts of transport processes take place in the proximal tubules [2], which are directly connected to the glomeruli. The basolateral Na^+^/K^+^ ATPase is responsible for setting up the electrochemical gradient for reabsorption in the proximal tubule. The concerted activity of these transport systems allows a vectorial movement of substrates resulting in the reabsorption of many important substances such as glucose from the filtrate into the blood, and the excretion of waste products such as creatinine and xenobiotics (drugs and their metabolites) from the blood into the urine [3]. Depending on the driving forces governing these transport processes, transporters are classified as solute carriers (SLCs), when their function is driven by the electrochemical gradient of the substrates and as ATP-binding cassette transporters (ABC transporters), when it is driven from the ATP hydrolysis [4]. Focusing on renal drug handling, the kidney is a key organ for their excretion [5]. It has been calculated that 32% of the top 200 prescribed drugs in the US are mainly eliminated by the kidneys [5]. However, detailed information on the expression of these transporters in human kidneys, as also a confirmation of the translational relevance of gender- and age-dependent transporter expression observed in experimental animals [6,7,8] is scarce.

For this reason we have measured the protein abundance of Multidrug Resistance Protein 1 (MDR1, P-Glycoprotein), Multidrug Resistance Associated Protein (MRP)1-4, Breast Cancer Resistance Protein (BCRP), Organic Anion Transporter (OAT) 1-3, Organic Cation Transporter (OCT) 2-3, Multidrug And Toxin Extrusion protein 1 (MATE1), Peptide Transporter (PEPT) 1-2 and Organic Cation Transporter-Like 2 (ORCTL2) (Table 1) by liquid chromatography-tandem mass spectrometry-based targeted proteomics in a set of human cortex kidney samples. Moreover, the protein expression of the transporter was related to age and gender of the donors and, for selected transporters, to the mRNA expression. OAT1 and 3, OCT2 and ORCTL2 were found to be the most abundant renal SLC transporters while MDR1, MRP1, and MRP4 were the dominating ABC transporters. Moreover, we found several significant correlations between different transporters, which may indicate their functional interplay in the basolateral uptake and apical excretion of drugs.

In the following, the functional organization in the kidneys of the transporters identified in this work is given. Transporters for organic cations are involved in the renal secretion of cationic substances, which is accomplished by an uptake from the blood into the proximal tubules cells followed by a transport from these cells into the urine. Organic cations are endogenous substances (neurotransmitters such as dopamine and histamine) and exogenous substances (drugs such as amiloride and metformin) [9]. Basolaterally localized OCT2 and 3 mediate the uptake of organic cations into the renal proximal tubules cells, while apically expressed MATE1 together with MDR1 mediate their excretion into the urine. While MATE1 function is stimulated by acidic pH in the primary urine, MDR1 required energy obtained from ATP hydrolysis [9]. Similarly, basolaterally expressed OAT1-3 mediate the cellular uptake of endogenous (several different substances such as cAMP and prostaglandin E_2_, for example) and exogenous (such as tetracycline and zidovudine, for example) organic anions [9] in exchange with monovalent (OAT2) or divalent (OAT1 and 3) organic anions [9]. The excretion of organic ions into the urine is then mediated in an ATP-dependent manner by MDR1, BCRP, and MRP4 expressed on the apical plasma membrane of proximal tubules cells [9]. 

The apically expressed ORCTL2 is probably involved in the transport of chloroquine and quinidine into the urine [10].

The interested reader can find an extensive description of renal transporters in dedicated reviews [9,11,12].

## 2. Results

**Figure 1:**
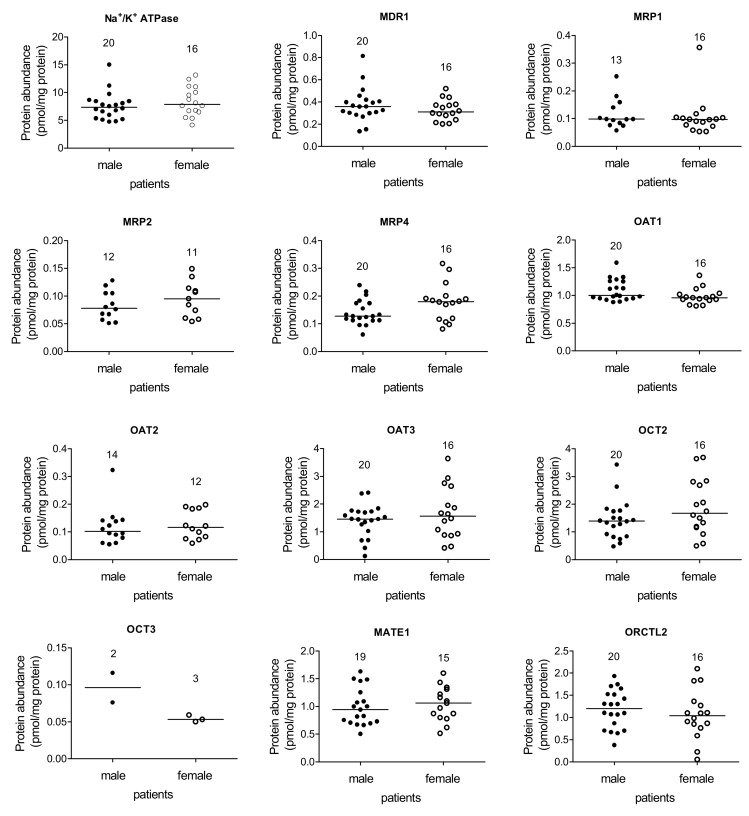
Transporter protein abundance (pmol/mg membrane protein) in samples of kidney cortices obtained from male (●) and female (○) patients. For none of the investigated transporters, a gender-dependent expression was detected (*p* > 0.05, unpaired *t*-test for normally distributed values and Mann-Whitney non parametric test for non-Gaussian distributed values (MRP1, OAT1 and 2)). The bars represent the median value of the respective transporter amount. The numbers indicate the number of patients for the group.

The first aim of this work was to measure the protein abundance of selected transporters, which play an important physiological and pharmacological role in human renal kidney cortices. While several basolaterally and apically localized transporters were well detectable, the protein abundance of MRP3, BCRP, PEPT1, and PEPT2 was below the lower detection limit of the proteomic assay. OCT3 was measurable only in few samples. Measurable protein amounts could be detected for Na^+^/K^+^ ATPase, MDR1, MRP1, 2, and 4, OAT1-3, OCT2, ORCTL2, and MATE1. Therefore, for these transporters it was evaluated, whether there was a gender-dependent protein expression. As shown in Figure 1, no gender-dependent expression at the protein level was detected.

**Figure 2:**
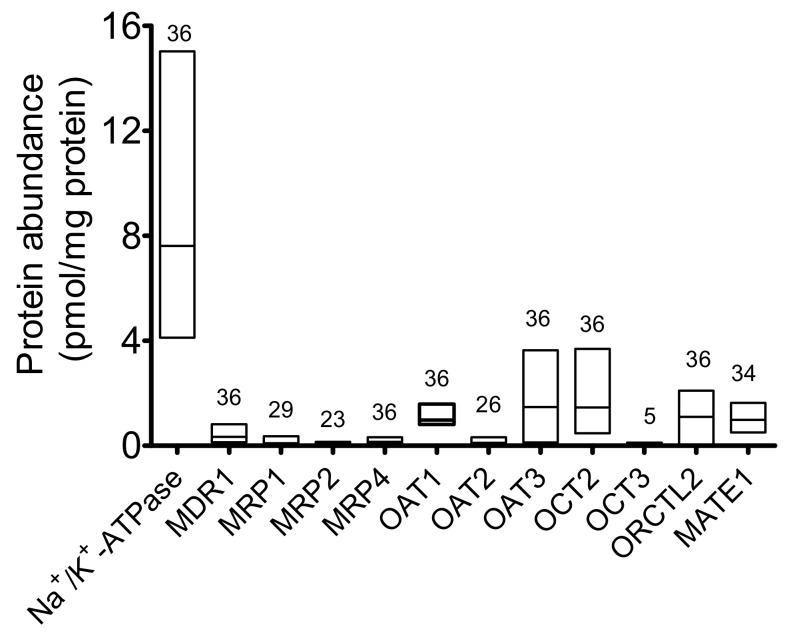
Transporter protein abundance (pmol/mg membrane protein) in the plasma membrane of samples from human kidney cortices (low-high bar with the median). Above the columns, the number of patients per group, where transporter abundance could be measured, is shown.

Therefore, the results obtained with samples derived from male and female patients were combined (Figure 2). As expected, the Na^+^/K^+^ ATPase showed high protein levels, followed by the SLC transporters OCT2 and OAT1 and 3. A discrete expression of MATE1 and ORCTL2 was also measured. ABC transporters, together with OAT2 and OCT3 were less abundant than the other proteins.

**Figure 3:**
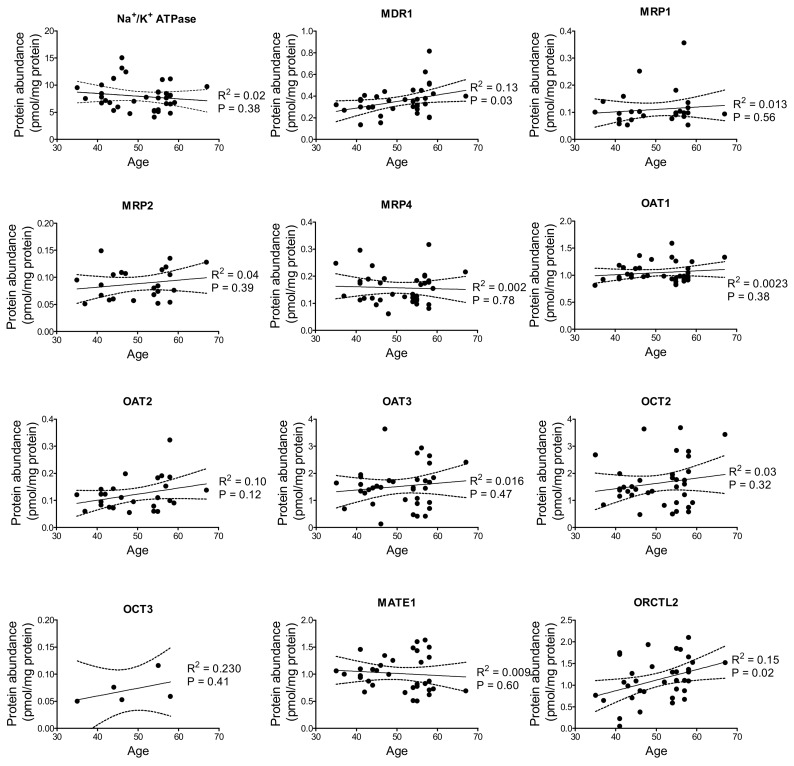
The relationship between transporter protein abundance (pmol/mg membrane protein) in samples of kidney cortices obtained from both male and female patients and their age. The linear regression line (solid line) with the 95% confidence interval (dashed lines) together with the coefficient of regression (R^2^) and the *p* value of the test whether the regression line is significantly different from zero are shown.

In an attempt to determine whether there is any association between the transporter protein abundance and the age of the donors, the protein data was stratified according to the age. We could observe a significant linear relationship between transporter protein abundance and age of donors for MDR1 and ORCTL2; in each case the transporter abundance increasing with age (Figure 3).

The analysis of relationships between transporter abundancy revealed several significant correlations (Table S1):

- Na^+^/K^+^ ATPase correlated directly with MRP2/MRP4/OAT2/OCT2 abundance;
- MDR1 with MRP2/OAT2/OAT3/OCT2;
- MRP2 with Na^+^/K^+^ ATPase/MDR1/MRP4/OAT3/OCT2;
- MRP4 with Na^+^/K^+^ ATPase/MRP2/OAT3/OCT2;
- OAT2 with Na^+^/K^+^ ATPase/MDR1/OAT3/OCT2/MATE1;
- OAT3 with MDR1/MRP2/MRP4/OAT2/OCT2/ORCTL2/MATE1;
- OCT2 with Na^+^/K^+^ ATPase/MDR1/MRP2/MRP4/OAT2/OAT3/ORCTL2/MATE1;
- OCT3 with MATE1 (inversely correlated);
- ORCTL2 with OAT3/OCT2;
- MATE1 with OAT2/OAT3/OCT2/OCT3

**Figure 4:**
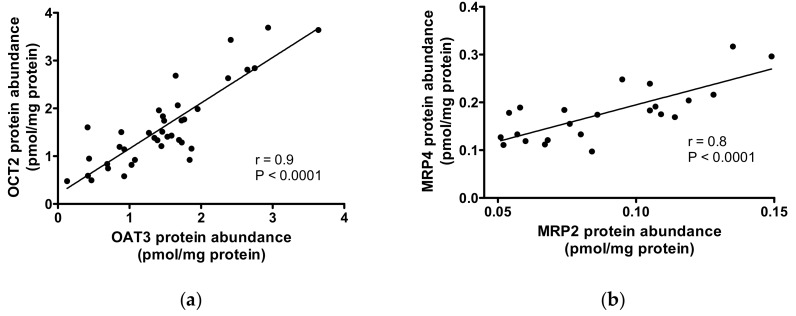
Correlations between transporter protein abundances. A strong correlation was observed between OAT3 and OCT2 abundance (panel (**a**)) and between MRP2 and MRP4 abundance (panel (**b**)). The Pearson correlation coefficient (*R*) and the *p* value of correlation significance are shown.

A strong correlation (Pearson *R* ≥ 0.8) was found between OAT3 and OCT2, and MRP2 and MRP4, abundance (Figure 4). Interestingly, the abundance of MRP1 and OAT1 was not significantly correlated to the expression of any other transporter, while OCT2 expression correlated with that of almost every other transporter.

Focusing on the secretion system for organic cations, consisting of basolaterally localized OCT2 and apically localized MATE1, only a weak but significant correlation could be found (*R* = 0.5, *p* = 0.005, Table S1). 

**Figure 5:**
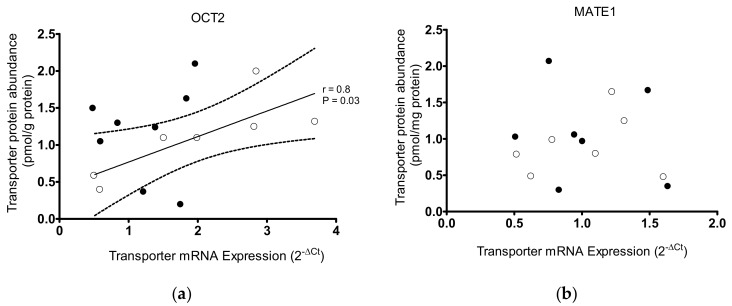

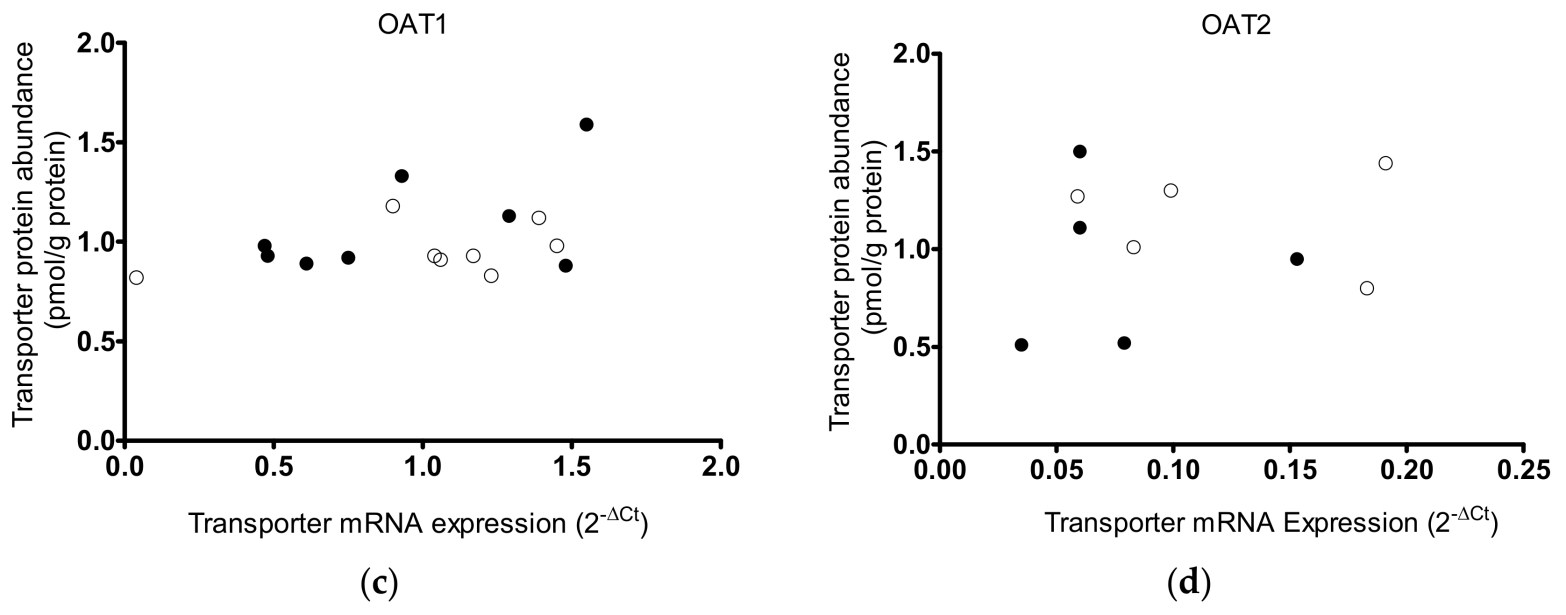
Relationship between mRNA expression and protein abundance for OCT2 (**a**), MATE1 (**b**), OAT1 (**c**), and OAT2 (**d**) in samples from kidney cortices from male (●) and female (○) patients. Only for hOCT2 in samples from female patients a strong linear relationship between mRNA and protein expression was observed, as evident from the Pearson correlation coefficient (*R* = 0.8) and the *p* value (= 0.03) of statistical significance of correlation. The linear regression line (solid line) with the 95% confidence interval (dashed lines) are shown. The mRNA content of these transporters is expressed relative to mRNA GAPDH expression as 2 ^−ΔCt^, as determined by real-time PCR analysis. Protein content measured by proteomic analysis was given as pmol transporter/mg membrane protein.

In addition, the mRNA expression of selected transporters (OCT2, OAT2, and MATE1) was measured in some patients by real-time PCR. Also, in this case, no correlation between mRNA-expression of the transporters and age and gender of the patients was detected (Figure S1). Interestingly, relating the mRNA expression and protein abundance of OCT2, MATE1, and OAT1 and 2 a linear correlation was only found for OCT2 in samples from female patients, suggesting that in male patients the protein abundance of OCT2 may be further influenced by post-translational modification (Figure 5a). For MATE1, OAT1 and OAT2 expression, no linear relationship between mRNA expression and protein abundance was detected (Figure 5b–d). 

**Figure 6:**
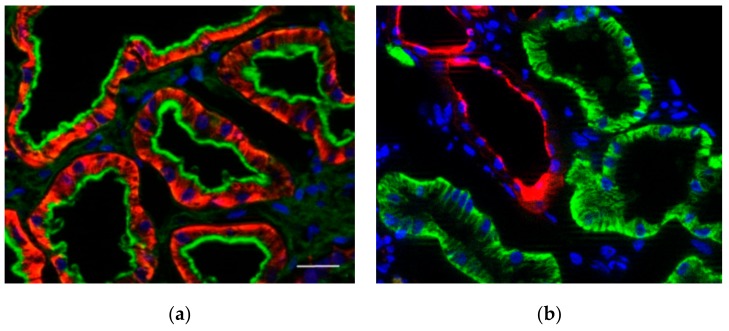
Localization of OCT2 in cortex of human kidneys. Panel (**a**) shows the OCT2 in red and the PHA-E labeling of proximal tubules in green. Panel (**b**) shows the OCT2 in green and the PNA labeling of distal tubules in red. The nuclei are labeled with DAPI (blue). OCT2 is exclusively localized in the basolateral membrane of proximal tubule cells. Scale bar = 20 µm.

Since the proteomic analysis revealed a substantial amount of OCT2 in the cortex of human kidneys, we performed an immunofluorescence analysis of renal OCT2 to confirm this finding. By co-labeling of proximal tubules with PHA-E (Figure 6a) and of distal tubules with PNA (Figure 6b), a high expression of OCT2 in the basolateral domain of the proximal tubules plasma membrane was evident, confirming OCT2 localization in this part of the nephron.

## 3. Discussion

The kidneys play an important role in maintaining body homeostasis. In order to execute this vital function, the renal tissue is equipped with a battery of membrane transporters, which are specifically expressed in well-defined apical and basolateral membrane domains, and which work together to attain vectorial movement of substrates through the renal tubules [3]. These processes can be tuned according to the pathophysiological situation. Therefore, knowledge of transporter abundance at protein level, of its dependence from gender and age and of its relationships with mRNA content in human kidneys is of great molecular, physiological, and pharmacological importance. For example, the interpretation of molecular and functional protein-protein interaction studies, of the interactions of exogenous and endogenous substances with renal transporters strongly profits from the determination of transporter expression profile. Such investigations became possible with the introduction of –omics technologies such as quantitative proteomics [23].

For this study, healthy parts of human renal cortices derived from tumor nephrectomy of adult patients were used. The samples were immediately processed after surgery.

The protein abundance was found here not dependent on sex. Similar results have been obtained with liquid chromatography-tandem mass spectrometry (LC-MS/MS) proteomics in kidney samples from Afro-Americans and Caucasians [24]. Taken together, these data show that there is a significant difference between humans and rodents. In fact, rodents are known to have a strong gender-dependent expression of some renal transporters [6,7]. This is of great importance to appreciate the translational relevance of animal experiments. 

As expected, the protein abundance of Na^+^/K^+^ ATPase resulted to be the highest under the transporters investigated in this study. In our opinion, this reflects the extreme importance of this pump, which contributes to maintain the gradients for Na^+^ and K^+^ between the extra- and intracellular space, being of primary importance for renal transport processes [25]. The transporters belonging to the SLC families (OCTs, OATs, MATEs) are more abundantly expressed at protein level in the membranes from human renal cortices than transporters belonging to the ABC-family, with exception for the Na^+^/K^+^ ATPase. The rank order of the most abundant renal drug transporters was OAT3 ~ OCT2 > OAT1 ~ ORCTL2 > MATE1 >MDR1 (P-gp). Similar findings have been obtained in other works [26,27], even though a direct quantitative comparison of these results is difficult because the data from different groups are often expressed in different units, such as pmol transporter protein /g renal tissue [26] or pmol transporter protein /mg membrane proteins [27]. However, the OCT2 results in every study to be highly expressed at protein level [24,26,27,28].

The protein abundance of OCT2 was positively correlated with the expression of almost all transporters investigated in this study, while MRP1 and OAT1 were not correlated to any other transporter. The strongest correlations were observed for transporters expressed on the same membrane domain: OCT2/OAT3 (basolateral membrane) and MRP2/MRP4 (apical membrane), suggesting that there may be a membrane domain specific regulation mechanism of protein expression. Interestingly, the correlations between proteins that act as functional counterparts for renal excretion at the basolateral and the apical membrane (e.g. OAT3 - MRP2 (excretion of anions), OCT2 – MDR1 (P-gp) and OCT2 – MATE1 (both excretion of cations) even though significant were rather weak (correlation coefficient (*R*) = 0.4–0.5). This was a somewhat unexpected finding as there are many well-documented examples for the net excretion via the respective transporters tandems (e.g. metformin [29] and trospium [30] via OCT2 and MATE1). A negative correlation was observed for OCT3/MATE1. However, these data are to be interpreted with caution, since only in few samples OCT3 protein amount was measurable. 

With respect to age-related differences in the protein abundance, only for MDR1 and ORCTL2 weak but significant associations could be observed, suggesting that at least in an age range between 35 and 70 years, transporter protein expression stays mainly at the same level, as also found in [24]. 

Focusing on the organic cation renal secretion axis, which is represented by basolateral OCT2 and apical MATE1, the mRNA expression of these transporters was investigated and was found not to change in relationship to age and sex. Also, the mRNA expression of OAT2, another representative of the SLC family, was not dependent on sex and age of the patients.

Correlating protein to mRNA expression, it was evident that mRNA abundance is not predictive for the amount of protein expressed, except for OCT2 expression in samples from female patients. For this reason, it can be speculated that post-translational mechanisms may be involved in determining the level of OCT2 protein expression in kidneys from male patients. It is also possible that other factors, such as drug treatment may affect transporter mRNA expression and/or protein abundance, as already described for ABC transporters [31]. Moreover, epigenetic regulation by miRNAs or DNA methylation may also contribute to the described lack of correlation between gene and protein expression [32]. 

The immunofluorescence analysis of OCT2 expression in human kidneys confirmed the high expression of this transporter in the basolateral domain of proximal tubules cells, as already demonstrated [21], corroborating the findings obtained with our proteomic analysis.

In conclusion, in this study a relative high number of freshly isolated human kidney cortex from adult patients was analyzed by quantitative targeted proteomics to determine transporter abundance in the plasma membrane. This approach allowed to investigate the relationship between protein abundance and age and sex of the patients. The SLC transporters resulted to be highly expressed, underlining the importance of renal secretion processes in the kidney cortex. Importantly, a gender-dependent correlation between protein and mRNA transporter expression was observed, suggesting that sex can influence the post-translational OCT2 processing.

## 4. Materials and Methods

### 4.1. Human Kidneys

**Figure 7:**
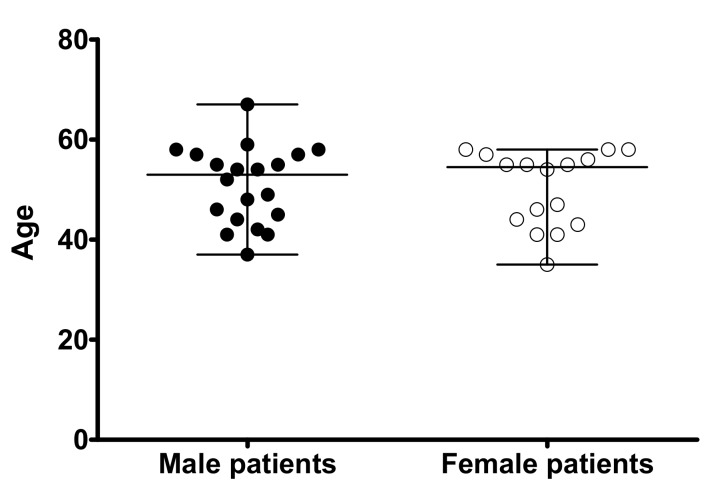
Age distribution of the male (*n* = 20) and female (*n* = 16) patients from which derive the kidney samples used in this study. The age of every single patient at the time of surgery together with the median and the range are shown (median 53 years, maximum 67 years, minimum 37 years and median 55 years, maximum 58 years, minimum 35 years for male and female patients, respectively). The median values were not significantly different (*p* > 0.05, Mann-Whitney non parametric test).

Human kidney samples were obtained from Caucasian patients (20 males and 16 females; median age 53 and 55 years, respectively, Figure 7) undergoing tumor nephrectomy in the Clinic for Urology of the University Hospital Münster. The procedure was approved by the local ethics commission (Approval number: 2008-030-f-S) and written consent was obtained from all patients. Immediately after nephrectomy, a piece of normal kidney tissue far away from the tumor was withdrawal and transferred into chilled HCO_3_^−^–free phosphate buffer. After this, the kidney cortex was isolated and cut into small pieces, which were placed into RNAlater solution (Qiagen, Hilden, Germany), or immediately frozen in liquid nitrogen, or fixed in 4% paraformaldehyde (Roth, Karlsruhe, Germany) for PCR, protein, and immunochemistry analysis, respectively.

### 4.2. Protein Quantification by LC-MS/MS

Sample preparation and protein quantification of MDR1 (P-gp, ABCB1), MRP1 (ABCC1), MRP2 (ABCC2), MRP3 (ABCC3), MRP4 (ABCC4), BCRP (ABCG2), PEPT2, OCT1, OCT3, OCTN2, OAT1, OAT2, OAT3, MATE1, OATP1A2, OATP1B1, OATP1B3, OATP2B1, NTCP and ASBT were measured by mass spectrometry-based targeted proteomics using validated LC−MS/MS methods as described elsewhere [33]. In brief, about 50 mg tissue frozen tissue were pulverized and the membrane protein fraction was extracted using the ProteoExtract Native Membrane Protein Extraction kit (Merck KGaA, Darmstadt, Germany) according to the manufacturer’s protocol. The obtained membrane fraction was subjected to determination of the whole protein concentrations using the bicinchoninic acid (BCA) assay (Thermo Fisher Scientific, Schwerte, Germany). If necessary, membrane fractions were adjusted to a maximum protein amount of 2 mg/mL. Subsequently, 100 µL of each membrane fraction were mixed with 10 µL dithiothreitol (200 mM, Sigma-Aldrich, Taufkirchen, Germany), 40 µL ammonium bicarbonate buffer (50 mM, pH 7.8, Sigma-Aldrich), and 10 µL ProteaseMAX (1%, m/v, Promega, Mannheim, Germany) and incubated for 20 min at 60 °C. After cooling down, 10 µL iodoacetamide (400 mM, Sigma-Aldrich) were added and the samples were incubated in a darkened water quench for 15 min at 37 °C. For protein digestion, 10 µL trypsin (trypsin/protein ratio: 1/40, Promega) was added and samples were incubated in a water bath for 16 h at 37 °C. Digestion was stopped by addition of 20 µL formic acid (10% *v/v*, Sigma-Aldrich). Afterwards, the samples were centrifuged one more time for 15 min at 16,000 g and 4 °C. 50 µL of the supernatant were mixed with 25 µL isotope-labeled internal standard peptide mix (10 nM of each labeled peptide, Thermo Fisher Scientific). All sample preparation and digestion steps were performed using Protein LoBind tubes (Eppendorf, Hamburg, Germany). Protein quantification was conducted on a 5500 QTRAP triple quadrupole mass spectrometer (AB Sciex, Darmstadt, Germany) coupled to an Agilent Technologies 1260 Infinity system (Agilent Technologies). Transporter proteins considered to be of clinical relevance were simultaneously quantified using proteospecific peptides (Table S2). Accuracy (error) and precision (CV) during sample analysis were both below 20%. Final protein abundance data (picomoles per milligram) were calculated by normalization to the total protein content of the isolated membrane fraction as determined by the BCA assay. All samples were digested and measured in duplicate.

### 4.3. Real-Time PCR Analysis

Human renal cortical tissue was homogenized, and total RNA was isolated using the RNeasy Mini Kit (Qiagen). RNA was separated using a RNeasy column (Qiagen) and for cDNA synthesis, 2 µg total RNA was used with the SuperScript-III First-Strand Synthesis SuperMix (Invitrogen, Karlsruhe, Germany). Gene expression profiles for OCT2, OAT1-2, and MATE1 were analyzed by real time PCR using SYBR Select Master Mix for CFX (Thermo Fisher, Waltham, MA, USA) on a CFX Realtime Detection System (Biorad, Hercules, CA, USA). Specific primer pairs as listed in Table 2 were used. Relative gene expression values were evaluated with the 2^-ΔCt^ method [34] using GAPDH as housekeeping gene. GAPDH mRNA expression in human kidneys seems not to be dependent on age, ethnicity, or gender [35].

### 4.4. Immunochemistry

Localization of OCT2 in human kidneys was investigated using specific antibodies raised in mouse against OCT2 [36]. Five µm thick cryosections were permeabilized with 0.2% Triton X-100 in PBS for 3 min, washed with PBS and then incubated for 1 h at room temperature in PBS containing 10% BSA (pH 7.4). After this, sections were incubated overnight at 4 °C with primary antibodies against OCT2 [36] at a dilution of 1:100. Proximal and distal tubules were identified by contemporaneously staining with phytohaemagglutinin (PHA-E, 1:200 in PBS, green fluorescence) or peanut agglutinin (PNA, 1:100 in PBS, red fluorescence), respectively (Biologo, Kronshagen, Germany) [37].

After washing in PBS, cryosections were incubated for 1 h at room temperature with secondary antibodies (Alexa Fluor 488 for green fluorescence or 594 for red fluorescence goat anti-mouse-Ig, Invitrogen, 1:1000) diluted in PBS, rinsed with PBS, coverslipped with FluoroMount (Sigma) and evaluated by epifluorescence microscopy (Observer Z1 with Apotome, Zeiss). Negative control slides were included without addition of primary antibody.

## Figures and Tables

**Table 1:** Overview of the transporters as investigated by proteomic analysis in this work.

Gene Name	Protein Name	UniProtKB ID	Plasma Membrane Domain
*ABCB1*	MDR1 (P-gp)	P08183	apical [13]
*ABCC1*	MRP1	P33527	basolateral [14]
*ABCC2*	MRP2	Q92887	apical [15]
*ABCC3*	MRP3	O15438	basolateral [16]
*ABCC4*	MRP4	O15439	apical [17]
*ABCG2*	BCRP	Q9UNQ0	apical [18]
*ATP1A1*	Na^+^/K^+^ ATPase 1	P05023	basolateral [19]
*SLC15A1*	PEPT1	P46059	apical [20]
*SLC15A2*	PEPT2	Q16348	apical [20]
*SLC22A2*	OCT2	O15244	basolateral [21]
*SLC22A3*	OCT3	O75751	basolateral (presumed)
*SLC22A6*	OAT1	Q4U2R8	basolateral [19]
*SLC22A7*	OAT2	Q9Y694	basolateral [19]
*SLC22A8*	OAT3	Q8TCC7	basolateral [21]
*SLC22A18*	ORCTL2	Q96BI1	apical [10]
*SLC47A1*	MATE1	Q96FL8	apical [22]

*ABCB* = ATP Binding Cassette Subfamily B; *ABCC* = ATP Binding Cassette Subfamily C; *ABCG* = ATP Binding Cassette Subfamily G; *ATP1A1* = Sodium/potassium-transporting ATPase subunit alpha-1; *SLC* = Solute Carrier.; MDR = Multidrug Resistance Protein; MRP = Multidrug Resistance Associated Protein; BCRP = Breast Cancer Resistance Protein; Na^+^/K^+^ ATPase 1 = Sodium/potassium-transporting ATPase subunit alpha-1; PEPT = Peptide Transporter; OCT = Organic Cation Transporter; OAT = Organic Anion Transporter; ORCTL = Organic Cation Transporter-Like; MATE = Multidrug and Toxin Extrusion Protein.

**Table 2:** List of primer sequences used for real-time PCR.

Primer	Sequences (5′ → 3′)	
OCT2	Forward	GGA ATA GCA TGG TTG AGG ACC A
	Reverse	GGG GCT ATG ATT CCC CCA AAA
MATE1	Forward	AAG CTG GAG CTG GAT GCA GTC
	Reverse	CAG CAG AGG AGC AGG ACG AGC
OAT1	Forward	CAG ACA GCT GTG TCA GGG AC
	Reverse	GAA TGG GCA TCC ACT CCA CA
OAT2	Forward	CTG GTT GGG TAC CTG ATA CGG
	Reverse	CAA GTA CCT GTG GGC CTC TTT
GAPDH	Forward	CAA GCT CAT TTC CTG GTA TGA C
	Reverse	GTG TGG TGG GGG ACT GAG TGT GG

## Supplementary Materials

Supplementary materials can be found at https://www.mdpi.com/1422-0067/20/21/5303/s1, Figure S1: Expression of mRNA for OCT2 (a), MATE1 (b), OAT1 (c), and OAT2 (d) in samples from kidney cortices of male and female patients in dependence from the age in years at the time of surgery. These figures show the mRNA content of these transporters expressed in comparison to the mRNA GAPDH expression as 2 ^−∆Ct^ measured by real-time PCR analysis. No significant difference of mRNA expression in samples from male and female patients was detected (not shown). Therefore, both genders were represented together. The linear regression line (solid lines) with the 95% confidence interval (dashed lines) together with the coefficient of regression (R^2^) and the P value of the test whether the slope of the regression lines is statistical significantly different from zero are shown. No significant linear relationship between transporter mRNA expression and age was detected; Table S1: Correlation between transporter abundance. The Pearson correlation coefficient (*R*) and the *p* value of the correlation are shown; Table S2: Overview of used proteospecific peptides and the respective MS parameters.
