# Supplementary material for: Protein Abundance of Clinically Relevant Drug Transporters in The Human Kidneys
Source: Int J Mol Sci. 2019 Oct 24;20(21):5303. doi: 10.3390/ijms20215303 (PMC6862022; doi:10.3390/ijms20215303)
Supplement: Supplementary file 1 [file ijms-20-05303-s001.pdf]

**Supplementary Materials:**

**Table S1.** Correlation between transporter abundance. The Pearson correlation coefficient (*R*) and the *p* value of the correlation are shown.

| | **$Na^+/K^+$ATPase** | **ORCTL2** | **MDR1** | **MRP1** | **MRP2** | **MRP4** | **OAT1** | **OAT2** | **OAT3** | **OCT2** | **OCT3** |
|---|---|---|---|---|---|---|---|---|---|---|---|
| **ORCTL2** | - | | | | | | | | | | |
| **MDR1** | - | - | | | | | | | | | |
| **MRP1** | - | - | - | | | | | | | | |
| **MRP2** | r=0.6<br>P=0.003 | - | r=0.5<br>P=0.02 | - | | | | | | | |
| **MRP4** | r=0.5<br>P=0.002 | - | - | - | r=0.8<br>P<0.001 | | | | | | |
| **OAT1** | - | - | - | - | - | - | | | | | |
| **OAT2** | r=0.4<br>P=0.046 | - | r=0.6<br>P=0.002 | - | - | - | - | | | | |
| **OAT3** | - | r=0.4<br>P=0.04 | r=0.4<br>P=0.01 | - | r=0.5<br>P=0.02 | r=0.5<br>P=0.003 | - | r=0.6<br>P=0.001 | | | |
| **OCT2** | r=0.4<br>P=0.008 | R=0.3<br>P=0.04 | r=0.4<br>P=0.02 | - | r=0.4<br>P=0.03 | r=0.5<br>P=0.001 | - | r=0.6<br>P=0.003 | r=0.9<br>P<0.0001 | | |
| **OCT3** | - | - | - | - | - | - | - | - | - | - | |
| **MATE1** | - | - | - | - | - | - | - | r=0.5<br>P=0.02 | r=0.6<br>P=0.0005 | r=0.5<br>P=0.005 | *r=-0.9*[1]<br>*P=0.04* |

[1] the values reported in Italic show a negative correlation between transporters expression

- indicates no significant correlation between transporters expression

**Table S2.** Overview of used proteospecific peptides and the respective MS parameters.

| Protein | Peptide | Q1 | Q3.1 | Q3.1-CE | Q3.2 | Q3.2-CE | Q3.3 | Q3.3-CE | DP | EP | CXP |
|---|---|---|---|---|---|---|---|---|---|---|---|
| P-gp | AGAVAEEVLAAIR | 635.4 | 971.4 | 27 | 430.2 | 23 | 900.3 | 27 | 80 | 10 | 13 |
| | AGAVAEEVLAAIR* | 639.5 | 981.4 | 27 | 440.2 | 23 | 910.3 | 27 | 80 | 10 | 13 |
| | IATEAIENFR | 582.4 | 749.4 | 30 | 565.2 | 28 | 678.4 | 28 | 70 | 10 | 13 |
| | IATEAIENFR* | 587.4 | 759.4 | 30 | 575.3 | 28 | 688.4 | 28 | 70 | 10 | 13 |
| MRP1 | DGAFAEFLR | 513.2 | 782.5 | 20 | 635.4 | 23 | 564.4 | 23 | 140 | 10 | 13 |
| | DGAFAEFLR* | 517.9 | 792.4 | 20 | 645.4 | 23 | 574.4 | 23 | 140 | 10 | 13 |
| MRP2 | LTIIPQDPILFSGSLR | 885.4 | 665.3 | 36 | 989.3 | 51 | 721.7 | 36 | 110 | 10 | 13 |
| | LTIIPQDPILFSGSLR* | 890.4 | 670.2 | 36 | 999.3 | 51 | 726.8 | 36 | 110 | 10 | 13 |
| | YLGGDDLDTSAIR | 698.5 | 547.3 | 43 | 1119.4 | 33 | 662.3 | 36 | 90 | 10 | 13 |
| | YLGGDDLDTSAIR* | 703.4 | 557.3 | 43 | 1129.4 | 33 | 672.3 | 36 | 90 | 10 | 13 |
| MRP3 | IDGLNVADIGLHDLR | 541.0 | 696.8 | 20 | 754.4 | 20 | 611.9 | 20 | 90 | 10 | 13 |
| | IDGLNVADIGLHDLR* | 544.3 | 701.8 | 20 | 759.3 | 20 | 616.8 | 20 | 90 | 10 | 13 |
| | HIFDHVIGPEGVLAGK | 563.9 | 749.5 | 27 | 650.4 | 29 | 513.4 | 37 | 151 | 10 | 13 |
| | HIFDHVIGPEGVLAGK* | 566.2 | 749.4 | 27 | 650.4 | 29 | 513.4 | 37 | 151 | 10 | 13 |
| MRP4 | SSLISALFR | 497.2 | 706.4 | 22 | 593.3 | 23 | 401.3 | 17 | 90 | 10 | 13 |
| | SSLISALFR* | 501.9 | 716.4 | 22 | 603.4 | 23 | 401.3 | 17 | 90 | 10 | 13 |
| BCRP | SSLLDVLAAR | 522.8 | 644.3 | 23 | 757.3 | 23 | 430.3 | 22 | 80 | 10 | 13 |
| | SSLLDVLAAR* | 527.9 | 654.3 | 23 | 767.3 | 23 | 440.3 | 22 | 80 | 10 | 13 |
| | VIQELGLDK | 507.3 | 802.5 | 20 | 674.5 | 23 | 545.5 | 30 | 120 | 10 | 13 |
| | VIQELGLDK* | 511.3 | 810.6 | 20 | 682.5 | 23 | 553.5 | 30 | 120 | 10 | 13 |
| MATE1 | QEEPLPEHPQDGAK | 525.7 | 594.9 | 21 | 615.4 | 31 | 650.6 | 24 | 140 | 10 | 13 |
| | QEEPLPEHPQDGAK* | 528.1 | 599.0 | 21 | 623.4 | 31 | 654.5 | 24 | 140 | 10 | 13 |
| | GGPEATLEVR | 514.94 | 457.9 | 23 | 617.3 | 23 | 688.4 | 30 | 65 | 10 | 13 |
| | GGPEATLEVR* | 519.9 | 462.8 | 23 | 627.3 | 23 | 698.4 | 30 | 65 | 10 | 13 |
| Na/K-ATPase | LSLDELHR | 328.3 | 435.2 | 15 | 391.7 | 14 | 669.3 | 15 | 100 | 10 | 13 |
| | LSLDELHR* | 331.5 | 440.3 | 15 | 396.8 | 14 | 679.4 | 15 | 100 | 10 | 13 |
| OAT1 | LVGFLVINSLGR | 644,5 | 758,5 | 30 | 659,4 | 30 | 871,6 | 30 | 140 | 10 | 13 |
| | LVGFLVINSLGR* | 649,6 | 768,5 | 30 | 669,3 | 30 | 881,7 | 30 | 140 | 10 | 13 |

| | | | | | | | | | | | |
|---|---|---|---|---|---|---|---|---|---|---|---|
| **OAT2** | NVALLALPR | 483.2 | 753.5 | 20 | 569.4 | 20 | 682.6 | 21 | 80 | 10 | 13 |
| | NVALLALPR* | 488.2 | 763.6 | 20 | 579.4 | 20 | 692.5 | 21 | 80 | 10 | 13 |
| **OAT3** | VAVFNGK | 367.3 | 564.3 | 15 | 635.3 | 17 | 465.4 | 18 | 60 | 10 | 13 |
| | VAVFNGK* | 371.2 | 572.4 | 15 | 643.5 | 17 | 473.3 | 18 | 60 | 10 | 13 |
| **OCT2** | WLISQNK | 444.8 | 589.5 | 21 | 476.3 | 21 | 702.6 | 21 | 100 | 10 | 13 |
| | WLISQNK* | 448.5 | 597.3 | 21 | 484.4 | 21 | 710.4 | 21 | 100 | 10 | 13 |
| **OCT3** | GIALPETVDDVEK | 693.3 | 1031.3 | 25 | 516.2 | 28 | 805.2 | 38 | 120 | 10 | 13 |
| | GIALPETVDDVEK* | 697.3 | 1039.3 | 25 | 520.3 | 28 | 813.3 | 38 | 120 | 10 | 13 |
| **PEPT1** | GNEVQIK | 394.3 | 616.4 | 15 | 487.3 | 15 | 388.1 | 15 | 60 | 10 | 13 |
| | GNEVQIK* | 398.0 | 624.4 | 15 | 495.3 | 15 | 396.2 | 15 | 60 | 10 | 13 |
| | TLPVFPK | 401.2 | 587.4 | 15 | 391.2 | 24 | 490.3 | 23 | 70 | 10 | 13 |
| | TLPVFPK* | 405.2 | 595.4 | 15 | 399.2 | 24 | 498.2 | 23 | 70 | 10 | 13 |
| **PEPT2** | IEDIPANK | 450.3 | 657.4 | 19 | 429.2 | 27 | 786.5 | 20 | 110 | 10 | 13 |
| | IEDIPANK* | 454.1 | 665.4 | 19 | 437.3 | 27 | 794.6 | 20 | 110 | 10 | 13 |
| **ORCTL2** | TDAQAPLPGGPR | 590.2 | 693.3 | 27 | 764.5 | 27 | 487.2 | 23 | 160 | 10 | 13 |
| | TDAQAPLPGGPR* | 595.1 | 703.5 | 27 | 774.3 | 27 | 487.3 | 23 | 160 | 10 | 13 |

Peptides highlighted in grey were used for quantification; peptides without accentuation serves as qualifier (CE, collision energy; DP, declustering potential; EP, entrance potential; CXP, collision cell exit potential; Q, quadrupole). .

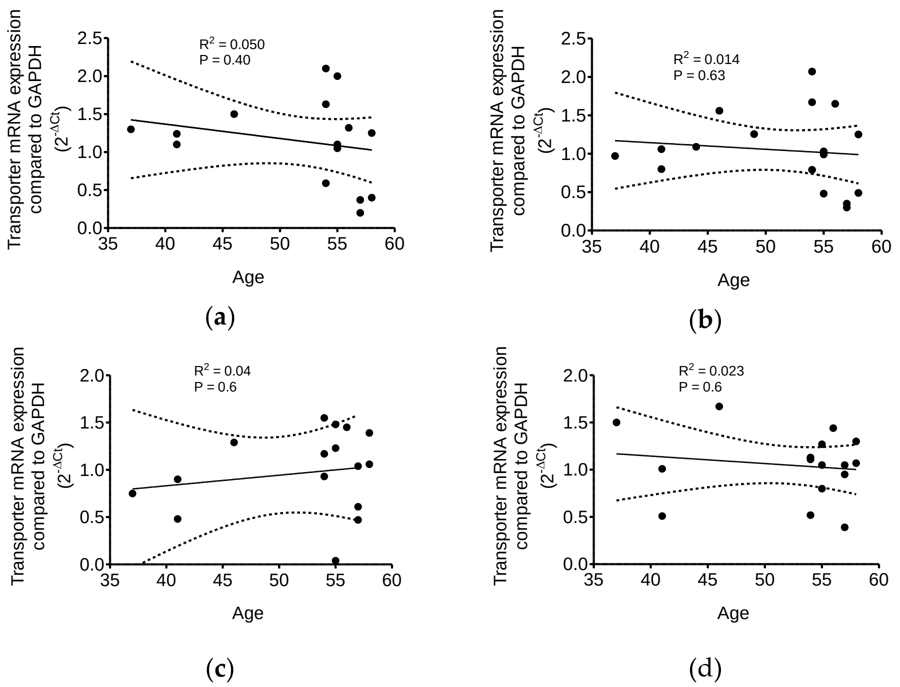


**Figure S1.** Expression of mRNA for OCT2 (**a**), MATE1 (**b**), OAT1 (**c**), and OAT2 (**d**) in samples from kidney cortices of male and female patients in dependence from the age in years at the time of surgery. These figures show the mRNA content of these transporters expressed in comparison to the mRNA GAPDH expression as $2^{-\Delta Ct}$ measured by real-time PCR analysis. No significant difference of mRNA expression in samples from male and female patients was detected (not shown). Therefore, both genders were represented together. The linear regression line (solid lines) with the 95% confidence interval (dashed lines) together with the coefficient of regression ($R^2$) and the P value of the test whether the slope of the regression lines is statistical significantly different from zero are shown. No significant linear relationship between transporter mRNA expression and age was detected.
